# Associations of carcass weight and trimming loss with cull dairy cow health observations collected at slaughter.

**DOI:** 10.1016/j.vas.2023.100285

**Published:** 2023-01-10

**Authors:** R. Vlemminx, M. Bouwknegt, B. Urlings, G. van Schaik

**Affiliations:** a Quality Assurance and Public Affairs department, Vion, Boxtel, The Netherlands; b Royal GD, Deventer, The Netherlands; c Faculty of Veterinairy Medicine, Utrecht University, Utrecht, The Netherlands

**Keywords:** Slaughter cattle, Carcass weight, Trimming loss, Postmortem health observations

## Abstract

Cull dairy cows account for around 27 percent of total head EU beef and veal production. For the Netherlands specific, even 42 percent (European Commission, 2022). As they are primarily kept to produce milk, red meat production is an additional source of revenue for dairy farmers. Insights in postmortem health observations that are not always visible on the living animal such as heart or liver issues, bruises, adhesions and injuries on the locomotor system, may contain valuable information for farmers to increase revenue and reduce losses in red meat production from cull dairy cows. Our goal was to obtain insights in the association of postmortem health observations with carcass weight and trimming losses. Data of 592,268 slaughter cows were available for analysis and models were built to explain carcass and trimming loss by the postmortem health observations. Carcass weight is lower for younger cows (-3.2 to -84.9 kg), cows with multiple health observations (-7.4 to -34.3 kg) and specific observations for the locomotor system (-16.7 to -22.7 kg), back (-17.9 kg), hindquarter (-21.6 kg) and chest and ribs (-15.5 to -27.6 kg). Total number of health observations (+2.0 to +6.5 kg), observations on the locomotor system (+3.3 to +5.4 kg) and on the chest and ribs (+2.2 to +9.8 kg) were the main predictors for trimming loss. Carcass weight is more affected by systemic health issues and diseases prior to slaughter leading to a negative energy balance and consequently reduced carcass weight. Trimming loss is more a consequence of the focus on meat quality and food safety in the slaughter process. Better understanding of the effect of on-farm management, on health, carcass weight and trimming loss will provide new insights for farmers and veterinarians but will also give them more action perspective to improve dairy farm preventive management and reduce losses at slaughter.

## Introduction

Dairy cows that go to slaughter account for around 27 percent of total EU beef and veal production. For the Netherlands even 42 percent of total head of slaughter cows are dairy cows (European Commission, 2022). Although contribution of dairy cows to beef production varies by country (Cabaraux et al., 2005), the sale of dairy cows accounts for a significant share of the total monetary revenue of dairy herds (Seegers et al., 1998). Dairy cows destined to be harvested for beef production are usually referred to as ‘cull dairy cow’ (Moreira et al., 2021b). “Culling” also can be used when cows leave the farm because of sale, salvage or death (Fetrow et al., 2006). In our paper, CDC refers to cows send for slaughter.

The revenue of a CDC is determined by carcass weight, conformation and fat, subtracted by trimmings resulting from health observations such as injuries, bruises, adhesions and inflammations. Carcass weight accounts for the carcass, so without head, skin, organs and shank. Carcass conformation score reflects shape and development of carcass measured by the SEUROP system. Carcass fat score represents the level of fat coverage on the outside and within the thoracic cavity of the carcass (Berry et al., 2021). As this determination is complex and not transparent, only few farmers understand the importance of beef quality and health observations on the ultimate value of their animals (Ahola et al., 2011).

Culling rate of Dutch dairy herds varies around 25% and more than 70% of the herds have an average culling rate of less than 30% (Nor et al., 2014). Among the most common culling reasons reported are fertility (problems), udder health, injury, disease and lameness of which especially the latter three can have a negative effect on carcass weight (Hadley et al., 2006; Moorman et al., 2018; Seegers et al., 1998). Some health issues, e.g. heart or liver issues can decrease CDC revenues, without farmers being aware of it. While the losses due to involuntary replacement of a cow varies from $235 to $650 depending on culling reason. Health related problems reported gross profit losses between $599 and $908 compared to healthy cows (Puerto et al., 2021a; b

). Sick cows have 2% less dressing percentage resulting in lower carcass values compared to healthy cows (Vogel et al., 2011). Heifers treated multiple times for bovine respiratory disease are smaller sized and have decreased growth rates and marbling scores (Montgomery et al., 2009).

Increased culling age has a positive effect on body size and carcass weight (Gallo et al., 2017). Carcass weight of first lactation CDC is less than those of multiparous cows with highest weights at lactation numbers 4 to 6 and decreasing from 7^th^ lactation onwards (Seegers et al., 1998). Conversely, cows culled at a younger age have the potential to be graded higher and have better carcass value. As culling rate is normally closely related to number of replacement heifers, an increase will have negative economic and environmental consequences. The average age of Dutch CDC is quite stable around 5.9 years (S.D. = 0.8) (Nor et al., 2014; Moreira et al., 2021b). However, herds with a higher longevity did not economically outperform herds with a lower longevity (Vredenberg et al., 2021).

Much research has been conducted on milk production and its relation with genetics, health disorders, culling reasons, longevity and economics. Recently, Collineau et al. (2022) analyzed a large dataset of French slaughter cattle on condemnation rates and underlying reasons. However, this mainly concerned beef cattle. Interestingly, sources of variation in beef production from CDC and its relation with carcass weight, trimming loss and health observations have rarely been investigated. We used a large dataset with carcass characteristics of 592,268 Dutch cows slaughtered in 2016-2020 in a slaughterhouse primarily processing dairy cows (>95%).

Our goal was to investigate the association between postmortem health observations such as injuries, bruises, adhesions and inflammations and carcass weight and trimming losses of CDC. This may provide new insights in sometimes hidden health issues (e.g. on heart or liver) that can influence carcass weight and trimming loss. This knowledge can be used to improve health management of dairy herds, reducing the risk of cows becoming diseased or injured and thus improve beef revenues of CDC.

## Materials and methods

### Data collection

**Fig. 1:**
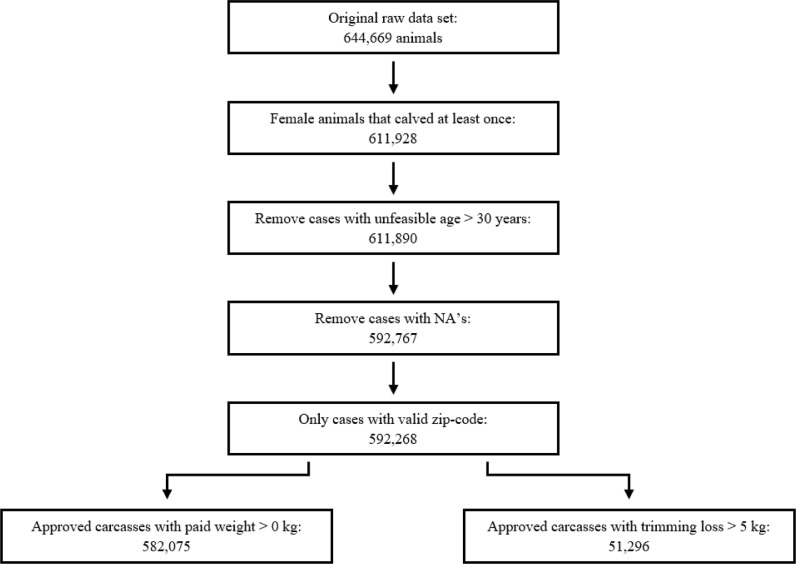
Data editing steps, starting with the raw dataset till the final datasets with approved carcasses by carcass weight and trimming loss > 5 kg of approved carcasses from Dutch cull dairy cows, 2016 – 2020.

A dataset of 592,268 female adult cattle (parity ≥ 1) from a single slaughterhouse located in the South of the Netherlands was used (Fig. 1). Cattle were slaughtered between January 1, 2016 and December 31, 2020. Cattle underwent antemortem (AM) and postmortem (PM) inspection by official veterinarians or official assistants (referred to as “meat inspection” from this point onward). The meat inspection was performed according to the specific rules for official controls on products of animal origin laid down in Regulation (EC) 854/2004 of the European Parliament. These include a set of activities before and after stunning, ante and post mortem (AM/PM) inspection involving visual inspection, palpation and incision of particular organs and lymph nodes. The dataset comprised farm of origin, animal identification, age of the animal, carcass weight, trimming loss and health observations registered during meat inspection.

### Dependent variables

Only carcass weights (in kg) from approved carcasses were taken into account, condemned carcasses were omitted from the dataset. Carcass weight, trimming loss and health observations were defined during PM-inspection according to the delegated EU Regulation 2017/1182 and the implementing EU Regulation 2017/1184. Trimming loss (in kg) was defined by amount of carcass meat trimmed during slaughter. Carcass weight and trimming loss excluded head, skin, organs and shank. Both variables are relevant for farmers as they get paid by carcass weight and cut by trimming loss. This paper primarily focusses on outcomes decreasing carcass weight by more than 10 kg or trimming loss exceeding 10 kg. This because they impact carcass revenue the most, making them relevant for herd management improvements.

### Independent variables

Several variables were available for further analysis. The slaughter age of cows was stratified to different age categories, i.e.: less than 1.5 years, 1.5 up to 2.5 years, 2.5 up to 3.5 years, 3.5 up to 5 years, 5 up to 7 years, and 7 and more years. Health observations recorded during PM inspection comprised 89 unique findings to be classified into pathologies, fecal contamination and a diverse group of non-specific observations. Our analysis focused on pathological observations. Also observations that weren't monitored during the full period or rarely observed were left out of the analysis. This resulted in thirty health observations (Table 1) which were matched with and assigned to 12 PM categories as defined by Veldhuis et al., (2021). One extra category, chest and ribs, was added to the analysis, as it didn't match the 12 PM categories. Total count of health observations per carcass was also determined.

**Table 1:** Fourteen main post mortem (PM) health categories that covered thirty health sub-observations of Dutch cull dairy cows slaughtered in a Dutch slaughterhouse between 2016 and 2020 (Veldhuis et al., 2021).

Main PM-category (i.e. health observation)	Underlaying health sub-observations^2^	# sub-observations
Back^1^	Pathology^3^ back	1
Chest and ribs	Pathology ribs, chest	2
Gastro intestinal tract*	Pathology gastro intestinal tract	1
Heart*	Endocarditis, pathology heart, pericarditis	3
Hindquarter*	Pathology rump and buttock	1
Kidneys*	Condemned kidneys, pathology kidneys, white-spotted kidneys	3
Liver fluke*	Liver fluke	1
Liver other*	Abscesses, abnormalities, condemned livers, pathology liver	4
Locomotor system*	Pathology hip, hock, knee, shoulder, feet	5
Lungs*	Pathology lungs, pneumonia	2
Neck*	Pathology neck	1
Pleura or peritoneum*	Pathology pleura, pathology peritoneum	2
Condemned	Condemned carcass, all organs condemned	2
Udder*	Mastitis, pathology udder	2

⁎ Post mortem category as defined by Veldhuis et al. (2021)

⁎⁎ Sub-observations were registered by official veterinarians or assistants

⁎⁎⁎ Pathology refers to injuries, bruises, adhesions and inflammations

Unique farm number (UBN) was included as random variable to account for between-farm differences. Quarter of the year was added to adjust for seasonal differences (e.g. grazing, weather conditions), with 1^st^ and 4^th^ quarter of the year being proxy for the non-grazing winter period. The distance from farm to slaughterhouse can influence carcass characteristics and health findings, centroid distances from areal postal codes were added to the dataset (Veldhuis et al., 2021) and stratified by subsequent groups of 25 kilometers each. Trimming loss was split into two groups of ≤ 5 kg and above 5 kg. Only the latter is relevant for farmers as only trimming loss above 5 kg will cut the price farmers receive.

### Statistical analyses

Data of carcass weight and trimming loss were analyzed by using univariable and multivariable linear regression. Models were fitted by maximum likelihood and t-tests using Satterthwaite's method (Satterthwaite, 1941).

Models for estimating carcass weight and trimming loss were as follows:

$$y=\beta_{0}+\beta_{1}X_{1}+\ldots +\beta_{n}X_{n}+\left(1|\text{UBN}\right)+\varepsilon$$

with: y = the carcass weight or trimming loss in kg

β_0_ = the intercept

β_1_X_1_ + … + β_n_X_n_ = the regression coefficients (β_1,…,n_) of independent variables (X_1,…,n_)

(1 | UBN) = random herd effect e = random error

Initial independent variables for the full multivariable linear regression models were slaughter year, season, age category, distance category, total number of health observations and presence of all of the health observations for locomotor system, pleura or peritoneum, heart, liver fluke, liver other, lungs, gastro intestinal tract, neck, kidneys, chest and ribs, back, hindquarter and udder. For both carcass weight and trimming loss two separate models were built to reduce the risk of confounding. One with the total count of health observations and one with all of the specific health observations. For model estimation of carcass weight all cows (n = 582,075) were taken into account. As paid price to farmers was only cut when exceeding 5 kilograms of trimming loss, only cows with more than 5 kilograms of trimming loss were taken into account for the model estimation of trimming loss (n = 51,296). The model for trimming loss with a random herd effect did not converge because few herds had more than one cow with over 5 kg trimming loss in the study period.

For all independent variables, the lowest category was used as default, except for age category. The reference category for age was ‘5 up to 7 years’ representing the largest group of cows (30%) and corresponding with the most likely culling age of Dutch dairy cows (Vredenberg et al., 2021). Collinearity was checked by Pearson's correlation. When two variables were highly correlated (r > 0.5) the variable with the strongest correlation with the dependent variable was added to the model. The Akaike Information Criterion (AIC) was used for evaluating model fit to the data (Akaike, 1972, 1974). All data were analyzed using RStudio and models were built by LMER-function (lme4-package) and GLM.NB-function (MASS-package). All independent variables were initially forced into full models and backward selection was applied to obtain a final model in which all variables were significant (P < 0.05) or retained as a cofounder. Confounding was checked by removing the non-significant (P ≥ 0.05) independent variables one by one from the final model and checking whether the estimates of the remaining variables changed for more than 25%. In such case, the confounder was retained in the model.

## Results

**Fig. 2:**
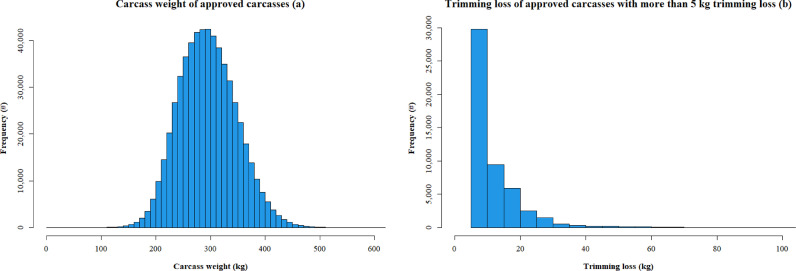
Histograms for carcass weight (n=582,075 cows) (a) and trimming loss (n=51,296 cows) >5 kg (b) of approved carcasses from Dutch cull dairy cows, 2016 – 2020.

Histograms of carcass weight and trimming loss are shown in Fig. 2. Dependent variables carcass weight and trimming loss were analyzed for normality by histograms and QQ-plots. Carcass weight showed normal distribution and trimming loss a logarithmic distribution. Table 2 contains all explanatory variables and the outcome of the univariable analysis for both dependent variables, i.e. carcass weight with a random farm effect and trimming loss. All explanatory variables were highly statistically significant (P < 0.001).

**Table 2:** Descriptive statistics^1^ for carcass weight and trimming loss for all explanatory variables of cull dairy cows slaughtered in a Dutch slaughterhouse between 2016 and 2020.

		Carcass weight (in kg, n = 582,075 cows)^2^					Trimming loss above 5 kg (in kg, n = 51,296 cows)^2^				
Characteristic	Category	% of carcasses	estimate	median	2.5% lower bound	97.5% upper bound	% of carcasses	estimate	median	2.5% lower bound	97.5% upper bound
Slaughter year	2016	20.7	293.6	291.4	198.6	399.2	19.4	12.7	9.2	5.1	38.4
	2017	24.4	290.3	287.2	197.0	394.2	20.7	11.6	8.2	5.1	34.7
	2018	22.3	292.0	288.6	200.8	396.2	19.4	11.6	8.0	5.1	33.6
	2019	14.3	302.0	300.4	202.8	412.7	15.2	13.5	9.4	5.2	46.0
	2020	18.3	305.7	303.8	205.0	416.4	25.3	13.2	9.3	5.2	44.4
Season	Winter	26.5	299.6	297.2	202.4	405.5	24.2	12.0	8.7	5.1	36.0
	Spring	22.3	296.3	293.2	199.2	403.5	21.9	12.2	8.6	5.2	39.8
	Summer	24.9	291.1	286.8	198.0	400.2	27.2	12.9	9.2	5.2	40.4
	Autumn	26.3	297.2	294.6	201.4	405.0	26.6	12.8	9.2	5.2	38.0
Age	< 1.5 years	0.0	223.2	219.0	152.3	357.4	0.0	10.0	10.0	10.0	10.0
	1.5 up to 2.5 years	8.4	264.0	262.4	171.2	359.8	5.1	13.0	8.6	5.2	48.3
	2.5 up to 3.5 years	12.9	278.1	273.0	187.7	381.8	8.3	12.5	8.5	5.2	48.0
	3.5 up to 5 years	26.0	300.4	297.6	204.6	406.2	21.0	12.1	8.5	5.1	40.0
	5 up to 7 years	30.0	306.6	302.8	212.2	412.6	34.3	12.4	9.0	5.2	36.8
	7 and more years	22.7	302.5	298.2	214.8	403.2	31.3	12.8	9.5	5.2	35.6
Distance to	< 25 kilometers	8.4	296.5	293.2	202.6	401.6	9.6	12.2	8.8	5.1	38.4
slaughterhouse	25 up to 50 km	17.3	295.3	294.0	201.5	406.2	18.5	12.5	9.0	5.2	39.0
	50 up to 75 km	18.5	297.0	292.4	201.2	404.2	19.5	12.8	9.0	5.2	40.4
	75 up to 100 km	19.7	295.9	291.0	196.7	401.2	18.9	12.5	9.0	5.2	38.0
	100 up to 125 km	12.3	296.3	293.0	200.0	401.2	11.0	12.4	9.0	5.2	37.5
	125 up to 150 km	7.6	295.4	293.8	199.4	404.0	6.8	12.4	9.0	5.2	37.8
	150 up to 175 km	8.5	297.3	296.6	203.2	405.6	8.2	12.2	9.0	5.2	35.8
	175 up to 200 km	5.3	295.3	294.0	199.8	404.0	5.0	12.6	9.0	5.2	37.8
	200 up to 225 km	2.4	292.7	292.4	199.6	410.2	2.5	12.8	9.2	5.2	42.0
Total health observations^3^	0	54.3	299.7	297.3	203.4	406.4	8.3	9.4	6.0	5.1	26.5
	1	27.1	294.2	292.0	198.7	401.9	42.6	11.7	8.8	5.2	33.8
	2	12.3	289.9	287.2	196.0	398.4	28.0	13.0	9.5	5.2	41.0
	3	4.5	286.4	284.2	194.9	394.6	13.4	14.3	10.0	5.2	47.2
	4	1.4	281.1	279.6	190.6	389.1	5.3	14.8	11.0	5.2	50.0
	5	0.4	277.1	275.0	188.6	382.4	1.8	15.	11.8	5.2	50.1
	≥ 6	0.1	271.6	273.0	188.0	365.4	0.6	16.5	11.8	5.3	59.5
Locomotor system^3^	0	93.9	297.1	294.4	201.0	404.2	63.3	11.2	8.0	5.2	37.0
	1	5.7	279.8	273.7	191.2	396.4	33.8	14.4	12.5	5.2	40.0
	≥ 2	0.4	271.9	263.0	185.1	387.3	2.9	17.1	14.0	5.2	47.4
Pleura or peritoneum^3^	0	87.3	296.6	293.8	200.8	404.6	83.3	12.7	9.2	5.2	40.0
	1	12.0	292.0	288.0	197.2	396.0	14.5	11.7	8.4	5.2	33.3
	2	0.7	290.6	287.2	192.4	405.4	2.2	10.4	7.0	5.2	32.6
Heart^3^	0	97.0	296.1	293.0	200.2	403.8	96.6	12.5	9.0	5.2	38.4
	≥ 1	3.0	295.9	293.2	199.9	404.0	3.4	13.0	9.4	5.2	40.1
Liver fluke^3^	0	94.7	296.1	293.2	200.4	404.0	95.4	12.5	9.0	5.2	38.6
	1	5.3	295.4	290.2	198.5	399.6	4.6	12.3	9.0	5.1	35.0
Liver other^3^	0	88.2	296.2	293.2	200.2	403.8	87.2	12.5	9.0	5.2	38.8
	1	11.6	295.3	291.6	200.9	403.0	12.6	12.3	9.2	5.2	36.8
	2	0.2	295.9	293.9	210.2	400.0	0.1	15.0	8.8	5.2	65.6
Lungs^3^	0	91.6	296.3	293.4	200.4	403.8	90.5	12.5	9.0	5.2	38.0
	1	8.4	293.4	289.2	197.8	402.8	9.5	12.7	9.2	5.2	41.9
Gastro intestinal tract^3^	0	98.0	296.1	293.2	200.3	403.8	97.6	12.5	9.0	5.2	38.3
	1	2.0	292.8	289.2	197.8	400.4	2.4	12.4	9.0	5.2	43.9
Neck^3^	0	98.1	296.1	293.2	200.1	403.8	94.3	12.5	9.0	5.2	38.4
	1	1.9	293.3	288.0	208.8	396.3	5.7	12.9	9.0	5.2	39.1
Kidneys^3^	0	95.0	296.6	293.8	200.4	404.2	91.3	12.5	9.0	5.2	39.0
	≥ 1	5.0	285.8	280.9	198.8	393.6	8.7	12.2	8.9	5.2	35.0
Back^3^	0	99.4	296.2	293.2	200.4	403.8	97.0	12.4	9.0	5.2	38.0
	1	0.6	275.6	267.2	190.8	383.8	3.0	15.6	9.9	5.2	60.0
Hindquarter^3^	0	94.5	297.2	294.6	201.6	404.4	57.9	11.7	8.1	5.2	32.0
	1	5.5	275.5	277.6	184.4	387.6	42.1	13.6	9.4	5.2	48.3
Chest and ribs^3^	0	97.5	296.5	294.0	200.6	404.0	85.7	12.2	9.0	5.2	36.0
	1	2.4	278.6	278.4	189.7	391.1	14.0	14.0	9.6	5.2	47.4
	2	0.1	263.6	273.2	177.5	361.3	0.3	23.3	14.5	5.3	99.5
Udder^3^	0	93.3	296.3	293.4	200.4	404.0	93.2	12.5	9.0	5.2	38.4
	1	6.6	292.5	288.2	199.5	397.6	6.7	12.4	8.8	5.1	39.0
	2	0.1	293.7	289.6	198.6	412.7	0.1	15.1	12.4	5.4	59.7

1 Unique farm number (UBN) as random effect

2 All variables P < 0.001

3 Number of health observations per category

The mean carcass weights in our study varied around 295 kilograms (S.D. = 52.8). The proportion of cattle slaughtered was lowest in 2019 (14%). Mean slaughter age was 5.4 years (S.D. = 2.3) and moderately skewed to the right (skewness = 0.73). The variation in carcass weight increased with slaughter age, except for oldest age category (Table 2). The majority of cows came from herds within 125 km distance of the slaughterhouse (76%) and the rest travelled a longer distance (24%). Half the cows had 1 or more post mortem health observations, of those most had 1 or 2 observations (80%). The most frequent health observations for carcass weight were ‘pleura or peritoneum’ (13%) and ‘liver other’ (12%). In case of trimming loss the most frequent health observations were ‘hindquarter’ (42%) followed by ‘pleura or peritoneum’ (17%), ‘chest and ribs’ (14%) and ‘liver other’ (13%). As shown in Table 2, frequency distributions of most variables were similar for carcass weight and trimming loss, except for ‘number of health observations’, ‘locomotor system’, ‘hindquarter’, ‘chest and ribs’ and ‘neck’.

### Multivariable regression models: carcass weight and trimming loss

The results of the final multivariable regression models for predicting carcass weight and trimming loss of approved carcasses are shown in Table 3. Observations on liver fluke (P = 0.751) were excluded from the model for carcass weight and observations on liver fluke (P = 0.514), lungs (P = 0.410) and gastro intestinal tract (P = 0.124) from the model for trimming loss. Based on univariate analyses herd effect accounted for less than 5% of total explained variation for trimming loss. The log-estimates and standard errors of trimming loss were converted to actual kilograms.

**Table 3:** Final multivariable regression models for carcass weight and trimming loss of approved carcasses of Dutch cull dairy cows slaughtered in a Dutch slaughterhouse between 2016 and 2020.

		Carcass weight (n = 582.075 cows)			Trimming loss^1^, above 5 kg (n = 51.296 cows)		
Independent variables	Category/value	Estimate (kg)	Std. error (kg)	P-Value	Estimate (kg)	Std. error (kg)	P-Value
Intercept^2^		315.7	1.03	< 0.001	8.8	1.02	< 0.001
Slaughter year (ref. = 2016)	2017	-4.3	0.19	< 0.001	-0.2	1.01	0.688
	2018	-2.3	0.19	< 0.001	-0.3	1.01	0.999
	2019	6.5	0.22	< 0.001	1.0	1.01	< 0.001
	2020	9.6	0.21	< 0.001	0.7	1.01	< 0.001
Quarter of the year (ref. = 1^st^)	2^nd^	-2.9	0.18	< 0.001	0.1	1.01	0.098
	3^rd^	-9.0	0.17	< 0.001	0.5	1.01	< 0.001
	4^th^	-2.9	0.17	< 0.001	0.5	1.01	< 0.001
Age category	< 1.5 years	-84.9	8.64	< 0.001	-1.9	1.92	0.634
(ref. = 5.0 up to 7.0 years)	1.5 up to 2.5 years	-43.5	0.24	< 0.001	0.7	1.01	< 0.001
	2.5 up to 3.5 years	-29.0	0.20	< 0.001	0.2	1.01	0.042
	3.5 up to 5.0 years	-6.5	0.16	< 0.001	-0.1	1.01	0.260
	7.0 and more years	-3.2	0.17	< 0.001	0.2	1.01	0.001
Distance category (ref. = 0 up to 25 kilometers)	25 up to 50 kilometers	0.0	1.18	0.978	0.2	1.01	0.724
	50 up to 75 kilometers	-0.5	1.19	0.694	0.3	1.01	0.826
	75 up to 100 kilometers	-2.3	1.13	0.045	0.1	1.01	0.044
	100 up to 125 kilometers	-2.4	1.15	0.034	0.1	1.02	0.344
	125 up to 150 kilometers	-3.5	1.23	0.005	0.1	1.02	0.807
	150 up to 175 kilometers	-2.7	1.16	0.023	-0.2	1.01	0.125
	175 up to 200 kilometers	-5.8	1.28	< 0.001	-0.0	1.01	0.006
	200 up to 225 kilometers	-8.7	1.59	< 0.001	-0.0	1.01	0.312
Number of health observations^4^	1	-7.4	0.14	< 0.001	2.0	1.01	< 0.001
(ref. = 0)	2	-12.3	0.19	< 0.001	3.1	1.01	< 0.001
	3	-16.6	0.30	< 0.001	4.4	1.01	< 0.001
	4	-22.5	0.52	< 0.001	4.9	1.02	< 0.001
	5	-26.9	1.01	< 0.001	5.5	1.02	< 0.001
	≥ 6	-34.3	2.01	< 0.001	6.5	1.03	< 0.001
Intercept^3^		316.0	1.02	< 0.001	8.0	1.01	< 0.001
Locomotor system (ref. = 0)	1	-16.7	0.26	< 0.001	3.3	1.01	< 0.001
	≥ 2	-22.2	0.97	< 0.001	5.4	1.02	< 0.001
Pleura or peritoneum (ref. = 0)	1	-6.0	0.19	< 0.001	0.1	1.01	0.028
	2	-12.6	0.71	< 0.001	-0.4	1.02	0.014
Heart (ref. = 0)	≥ 1	1.0	0.37	0.008	0.3	1.02	< 0.013
Liver other (ref. = 0)	1	-0.6	0.19	0.002	-0.2	1.01	0.015
	2	-0.1	1.51	0.940	1.6	1.08	0.002
Lungs (ref. = 0)	1	-3.1	0.23	< 0.001	not in model for trimming loss		
Gastro intestinal tract (ref. = 0)	1	-2.5	0.43	< 0.001	not in model for trimming loss		
Neck (ref. = 0)	1	-4.3	0.45	< 0.001	0.6	1.01	< 0.001
Kidneys (ref. = 0)	≥ 1	-11.3	0.28	< 0.001	-0.3	1.01	< 0.001
Back (ref. = 0)	1	-17.9	0.77	< 0.001	2.3	1.02	< 0.001
Hindquarter (ref. = 0)	1	-21.6	0.27	< 0.001	2.4	1.01	< 0.001
Chest and ribs (ref. = 0)	1	-15.5	0.39	< 0.001	2.2	1.01	
	2	-27.6	2.82	< 0.001	9.8	1.05	< 0.001
Udder (ref. = 0)	1	-1.6	0.25	< 0.001	0.1	1.01	0.387
	2	-1.0	1.89	0.595	1.6	1.09	0.035

1 Log-values of the estimates and standard error were transformed into actual kg.

2 Intercept for model with number of health observations

3 Intercept for model with specific health observations

4 Models were split for number of health observations and specific health observations, so estimates for number of health observations were calculated separately from estimates for specific health observations.

Adjusted R-squared values for carcass weight were 0.30 and 0.32 for the model with total number of health observations and all specific health observations. For the generalized linear models of trimming loss with total number of health observations and all specific health observations, pseudo R-squared were 0.04 and 0.11 respectively. ‘Age category’, ‘number of health observations’, ‘locomotor system’, ‘hindquarter’ and ‘chest and ribs’ explained most variance in carcass weight. ‘Number of health observations’, ‘locomotor system’ and ‘chest and ribs’ explained most variance in trimming loss. When the number of health observations increased, carcass weight decreased and trimming loss increased significantly.

## Discussion

Beef production of dairy cattle and the impact of specific health observations has received little attention in the scientific literature. With a large dataset (n=592,268) of Dutch slaughter cows, the association of post-mortem health observations with carcass weight and trimming loss (exceeding 5 kg) were determined. In our model, random herd effect explained around 20 percent of total variation in carcass weight, which is less compared to Klinger et al. (2021). They stated that prevalence of health observations can be explained for around 60 percent by agricultural holding and 20 percent by the slaughterhouse. However, their model contained less explanatory variables compared to ours.

Several of the health observations that substantially affect (>10 kg) carcass weight or trimming loss are relevant for dairy farm health management in terms of revenue and management options. Communicating slaughter data to farmers can help them to optimize their revenues from beef production.

As carcass weight and trimming loss define carcass value, both outcomes are relevant for improving economic revenues of dairy cows. Studies on the value of carcasses from dairy cows are scarce as its value is seldom taken into account (Gallo et al., 2017; Moreira et al., 2021b). Revenue of dairy cows is usually reported based on live weight and carcass weight or the ratio between the two (dressing percentage). In the Netherlands, cows are traded by intermediaries and transported to the slaughterhouse by third parties which all have to meet the same legal criteria. In practice, however, this means in most cases only monetary slaughter value and carcass weight are communicated to the farmer. Trimming loss and health observations are seldomly communicated to Dutch farmers and are thus invisible and unknown. In our study, no data on live weight were available and therefore we focused on carcass weight and trimming loss. Recent studies on CDC showed live weights (LW) around 640 kg and carcass weights ranging between 248 and 407 kg with most weights around 300 (S.D. = 35) kilograms (Seegers et al., 1998; Berry et al., 2021; Mehtiö et al., 2021; Moreira et al., 2021a; b). Our study showed similar outcomes for carcass weight with a mean of 295 kg (S.D. = 53). Trimming loss was treated as a separate outcome. In several studies ‘trimming loss’ is mentioned to affect overall value of a beef carcass and net loss of weight and product yield. In most cases, trimming losses are linked to superficial bruises on live animals and carcasses (Strappini et al., 2009; Paris et al., 2015; Eastwood et al., 2017; Kline et al., 2020). We focused on trimming loss caused by subsurface health observations affecting revenue of CDC similarly to carcass weight.

### Carcass weight

The type of culling decision is associated with the value of the culled cow, where it can be classified as either voluntary (economic) or involuntary (biological) (Moreira et al., 2021b). In the study period, involuntary culling was also due to legislative reasons. The initiation of Dutch phosphate legislation in 2017 led to phosphate reduction plans in the Dutch dairy sector and increased culling of cattle. Cows, calves and heifers accounted for total phosphate production at farm level. This led most farmers to first cut numbers of young stock to maintain milk production levels and, second, reduce number of cows when phosphate quota was still expected to be exceeded. This led to forced replacement of cows in 2017 and 2018 resulting in increased selection of young, early lactating cows and sale of young stock. Average slaughter age in 2017 and 2018 was up to half a year lower compared to other years. Total number of dairy cows and young stock dropped by 13% and 28% respectively. The average young stock rate per 10 cows declined from 7.3 to 6.1 (CBS, 2022). As shown in Table 3, lower age categories correspond with significant less carcass weight (-6.5 to -84.9 kg), which may explain different carcass weights over the years.

In our study, we found similar results as Seegers et al. (1998) for carcass weight and slaughter age, with carcass weight being highest between 5 and 7 years of age (306.6 kg). The relation between age and carcass weight was also described by others (Arendonk, Stokvisch, Korver, & Oldenbroek, 1984; Mehtiö et al., 2021; Veldhuis et al., 2021). By 2019, all herds were reduced and relative young stock numbers were low, resulting in fewer selection possibilities to CDC to be sent for slaughter. In 2019 and 2020, there was fewer young stock, so cows were kept longer to maintain farm level milk production leading to a shift towards more late lactation culling of cows. As shown by (Mehtiö et al., 2021), carcass weight drops in first month of lactation to increase from 2^nd^ month of lactation and onwards with similar results for 1^st^ to 5^th^ parity.

Seasonal differences were present with lowest carcass weights in summer (-9.0 kg). July to September account for highest average temperature and peak temperatures leading to increased heat-stress and impaired animal health with more risk of early lactation culling at lower body weight levels. As heat stress (Temperature Humidity Index > 68) during the full dry period reduces body weight gain, warm summer conditions may influence postpartum body weight and consequently slaughter weight as well (Fabris et al., 2019). Also grazing, mainly in spring and summer (the 2^nd^ and 3^rd^ quarter of the year), could reduce body weight and consequently carcass weight. Resulting from an energy deficit due to increased movement, decreased energy uptake from grass containing less energy combined with insufficient supplemented energy (Baudracco et al., 2012; Schären et al., 2016).

A negative association was observed between distance from farm to slaughterhouse and carcass weight, with reduced estimated carcass weight of 8.7 kg (2.8% of CW) for distances above 200 kilometers. Cattle that is transported has to maintain balance and has increased contact producing fatigue and bruises which may negatively affect carcass weight (Losada-Espinosa et al., 2018). Vogel et al. (2011) also reported increased body weight losses (up to 3.1%) and reduced muscle moisture for slaughter cows during marketing and transport. This may be caused by insufficient water and lack of feed intake during transport of slaughter cows. Increased storage of milk within the udder, in other words a shift of body fluids, as transport time increases might influence carcass weight as the udder is removed from a carcass prior to weighing it. Interestingly, distance categories were only associated with carcass weight and not with trimming loss (variation between -0.2 and +0.3 kg).

All health observations, except for heart health observations, had a negative impact on carcass weight. Major endocarditis combined with a second health observation is a reason for condemnation of the whole carcass. As condemned carcasses were not taken into account, the heart related reasons may be only mild cases which did not (yet) influence carcass weight. Increased number of health observations, regardless of type, resulted in lower carcass weights. Effect was highest for observations on ‘locomotor system’, ‘hindquarter’ and ‘chest and ribs’. All three are related to the musculoskeletal system of a cow, which may suggest that those health observations impact the amount of approved meat on a carcass most. A study of Seegers et al. (1998) showed results with lower carcass values for animals with health related culling reasons and heavier carcasses for reproduction and udder related culling reasons. In our study, we observed heavier carcasses for heart related health observations for which we have no explanation. Beaudeau et al. (1994) observed that culling in early lactation is more related to one severe disease compared to more complex culling decision making after 45 days post-partum. Our dataset lacked information to study cause and effect between culling reasons, lactation stage and health observations. Possibly, disease in early lactation affects the cow more than disease later in lactation. In early lactation, the cow is most vulnerable and often in a negative energy balance, which may explain lower slaughter weights at early lactation culling. Within our study, it was not possible to link the health observations at slaughter to the causal disease on the farm, which would be an interesting area for further research.

The effect on carcass weight of several health observations, e.g. locomotor system (-16.7 to -22.2 kg), back (-17.9 kg), hindquarter (-21.6 kg) and chest and ribs (-15.5 to -27.6 kg) is larger compared to other health observations (+1 to -12.6 kg). These high impact health observations also have a relatively high frequency of occurrence. Locomotor system and hindquarter are both part of the musculoskeletal system of the cow containing much muscle tissue and influencing the mobility of a cow. Reduced mobility affects feed uptake and consequently energy intake of the cow resulting in body weight loss. Alawneh et al. (2012) reported significant live weight loss from a single lameness episode averaging 61 kg. Lameness and injury to the hock and knee are leading welfare concerns affecting culling cow weight and thus economic value (Moreira et al., 2021b) resulting in indirect economic loss at culling (Edwardes et al., 2022).

### Trimming loss

Similar to carcass weight, cows culled in 2017 and 2018 had slightly lower trimming loss compared to 2019 and 2020. This may be explained by more forced culling of relatively healthy cows due to phosphate regulations. Overall, there is not much difference in trimming loss when considering season, age, distance to slaughterhouse and specific health observations. On the other hand, ‘total number of health observations’, single observations for ‘locomotor system’ or ‘chest and ribs’ were associated with increased trimming loss. A higher number of health observations probably indicated a poor general health that also resulted in rejecting more meat, for instance, meat close to the knee joint of the hind leg or rib-meat close to the lungs or chest. Health observations in the pleura or peritoneum interacted (P < 0.05) with several other health observations such as the locomotor system, lungs, liver fluke, neck, back, chest and ribs and hindquarter. This may indicate that pleura or peritoneum related health observations often coincide with other health observations leading to increased trimming loss. Insights in treatments of cows prior to their culling may provide relevant information to better understand causes of and interactions between specific health observations and their effect on trimming loss. Number and type of health observations may also differ per lactation stage as culling in early lactation is associated with one or more severe diseases compared to more complex culling decisions made in later stages of lactation (Beaudeau et al., 1994).

### Carcass weight compared to trimming loss

Although, total number of health observations, observations of the locomotor system or chest and ribs significantly impact carcass weight and trimming loss, farmers may experience them differently. The latter will be seen more as a real loss compared to the first because in the slaughter information (if shared) the trimming loss is depicted while a reduced carcass weight is not made explicit. This generates the question which insights should be provided to farmers when communicating slaughter information. Providing both expected and realized carcass weight and trimming loss will broaden their view. Our model for carcass weight provides a benchmark for carcass weight of comparable cows with and without postmortem health observations.

In contrast to other independent variables, the frequencies for ‘number of health observations’, ‘observations for locomotor system’, ‘hindquarter’ and ‘chest and ribs’ differ between the two dependent variables, i.e. carcass weight and trimming loss. These four health observations seemed to influence trimming loss more than other variables. Most ‘organ related’ health observations have similar distribution for zero, single and/or multiple health observations for carcass weight and trimming loss. This may indicate that carcass weight was more affected by systemic health issues and diseases prior to slaughter leading to a negative energy balance and consequently reduced carcass weight. Trimming loss is more a consequence of the focus on meat quality and food safety in the slaughter process and consequently trimming meat. For example, a cow with pneumonia may have lost a lot of body weight as a consequence of being diseased, resulting in reduced carcass weight with limited or no effect on trimming loss.

### Limits of this study

For the study, data from one slaughterhouse in het Southern part of the Netherlands was available. As shown by Veldhuis et al. (2021) and our study, distance to slaughterhouse does impact results. This should be taken into account when comparing our results with others. It is also known that performance and inter-official assistant variability are known challenges of meat inspection and bias apparent prevalence of meat inspection findings (Arzoomand et al., 2019). However, this bias is expected to be rather constant over time, thus meaningful trends may still be derived from meat inspection data. We have no information on which percentage of culled cattle per farm actually was delivered to the slaughterhouse nor do we know how representative the study population is for the total slaughter cattle population. Therefore, results per farm and for the total population have to be interpreted carefully. As voluntary culling is economically driven, market prices of milk and meat may influence the decision for farmers to cull a dairy cow and, consequently, the revenues at slaughter. In general, farm management of Dutch dairy farms is high-tech and intensive, from that perspective our results may be relevant for comparable high producing dairy farming systems in other temperate regions in the world.

## Conclusions

Carcass weight and trimming loss of cull dairy cows are associated with the total number of postmortem health observations and specific postmortem individual health observations. Carcass weight is considerably lower for younger cows, cows with multiple health observations and single observations for the locomotor system, back, hindquarter and chest and ribs. The total number of postmortem health observations and an observation for the locomotor system or chest and ribs were the main predictors for trimming loss. Differences between carcass weight, trimming loss and health observations and their associations with milk production, health observations and culling reasons of dairy cows are not fully understood. Better understanding of cause and effect of on-farm management, on health, carcass weight and trimming loss will not only provide new insights for farmers and veterinarians but will also give them more action perspective to improve dairy farm preventive management and reduce losses at slaughter.

## Declaration of Competing Interest

The authors declare the following financial interests/personal relationships which may be considered as potential competing interests: Rik Vlemminx reports a relationship with Vion Food Group that includes: employment. Martijn Bouwknegt reports a relationship with Vion Food Group that includes: employment. Bert Urlings reports a relationship with Vion Food Group that includes: employment. Gerdien van Schaik reports a relationship with Royal GD that includes: employment.
